# Gut microbiota is essential in PGRP-LA regulated immune protection against *Plasmodium berghei* infection

**DOI:** 10.1186/s13071-019-3876-y

**Published:** 2020-01-06

**Authors:** Li Gao, Xiumei Song, Jingwen Wang

**Affiliations:** 1 State Key Laboratory of Genetic Engineering, School of Life Sciences, Fudan University, Shanghai, 200438 People’s Republic of China; 2 Ministry of Education Key Laboratory of Contemporary Anthropology, School of Life Sciences, Fudan University, Shanghai, 200438 People’s Republic of China

**Keywords:** PGRP-LA, Gut microbiota, Peritrophic matrix, Immune effectors, *Plasmodium berghei*, *Anopheles stephensi*

## Abstract

**Background:**

Malaria remains to be one of the deadliest infectious diseases and imposes substantial financial and social costs in the world. Mosquitoes rely on the immune system to control parasite infection. Peptidoglycan recognition proteins (PGRPs), a family of pattern-recognition receptors (PRR), are responsible for initiating and regulating immune signaling pathways. PGRP-LA is involved in the regulation of immune defense against the *Plasmodium* parasite, however, the underlying mechanism needs to be further elucidated.

**Methods:**

The spatial and temporal expression patterns of *pgrp-la* in *Anopheles stephensi* were analyzed by qPCR. The function of PGRP-LA was examined using a dsRNA-based RNA interference strategy. Western blot and periodic acid schiff (PAS) staining were used to assess the structural integrity of peritrophic matrix (PM).

**Results:**

The expression of *pgrp-la* in *An. stephensi* was induced in the midgut in response to the rapid proliferating gut microbiota post-blood meal. Knocking down of *pgrp-la* led to the downregulation of immune effectors that control gut microbiota growth. The decreased expression of these immune genes also facilitated *P. berghei* infection. However, such dsLA treatment did not influence the structural integrity of PM. When gut microbiota was removed by antibiotic treatment, the regulation of PGRP-LA on immune effectors was abolished and the knock down of *pgrp-la* failed to increase susceptibility of mosquitoes to parasite infection.

**Conclusions:**

PGRP-LA regulates the immune responses by sensing the dynamics of gut microbiota. A mutual interaction between gut microbiota and PGRP-LA contributes to the immune defense against *Plasmodium* parasites in *An. stephensi*.
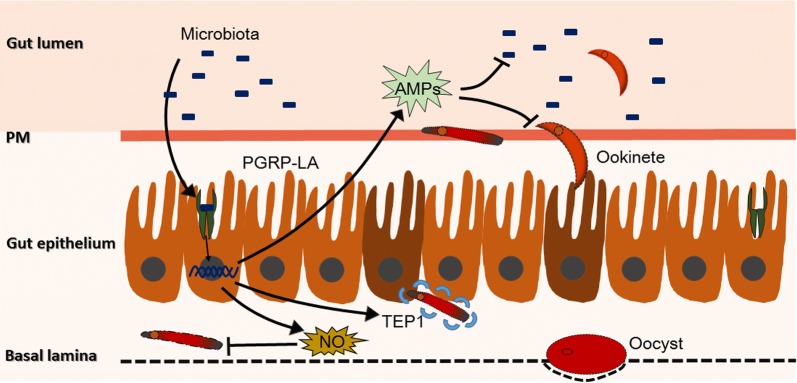

## Background

Malaria, a mosquito-borne disease (MBD), is caused by parasites of the genus *Plasmodium*. It remains to be a high concern of the World Health Organization due to the continued emergence and spread of drug-resistant parasites and insecticide-resistant mosquitoes [1]. Malaria prevention and control primarily relies on *Anopheles-*targeted intervention strategies [2]. Consequently, a further understanding of the interaction between the *Anopheles* mosquito and *Plasmodium* is extremely urgent.

The main bottleneck for *Plasmodium* infection in the mosquito is the traverse of ookinetes across the midgut [3, 4]. During this process, two physical barriers are encountered by *Plasmodium*. The first barrier, peritrophic matrix (PM), composed of chitin, proteoglycans and proteins, is produced by intestinal epithelial cells in response to blood-feeding [5, 6]. PM poses an indispensable role in the defense of *Plasmodium* because its maturation time coincides with the ookinete invasion time [7]. When artificially increasing the thickness of PM by feeding mosquitoes with latex particles and animal blood, the number of *P. gallinaceum* oocysts significantly decreases in *Aedes aegypti* [8]. Midgut epithelium is the second barrier that inhibits *Plasmodium* infection [9]. When ookinetes start to traverse the midgut epithelium, epithelial nitration will be activated, promoting thioester-containing protein 1 (TEP1)-mediated lysis of *Plasmodium* [10, 11]. Once inside the cell cytoplasm, the invaded intestinal epithelial cells tend to undergo apoptosis that extrudes ookinetes from the epithelium [7, 12]. Besides, epithelial cells are also immune competent cells, involved in the production of nitric oxide (NO), antimicrobial peptides (AMPs) and reactive oxygen species (ROS) to limit *Plasmodium* survival [13, 14].

Mosquito gut microbiota is another important factor that can influence the outcome of *Plasmodium* infection [15–19]. Oral administration of live or heat-inactivated bacteria isolated from mosquito midgut significantly decreases the infection rate of *P. falciparum* [20]. *Wickerhamomyces anomalus*, a yeast involved in symbiotic relationships with *Anopheles*, is able to directly eliminate *Plasmodium* through secreting killer toxin [21, 22]. Another stably inherited gut commensal bacteria, *Asaia*, can be genetically modified and directly inhibits pathogen development by secreting antiplasmodial effectors [23]. In addition, these microbes inhibit parasites indirectly through promoting the formation of PM and stimulating the oxidative defense system and NF-κB dependent immune responses [24–27].

Mosquito immune response plays a pivotal role in protecting the host against pathogen infection. Peptidoglycan recognition proteins (PGRPs) are a family of important pattern-recognition receptors (PRR) that initiate immune signaling pathways [28, 29]. In *Anopheles gambiae*, PGRP-LC is the receptor of NF-κB transcription factor REL2-mediated signaling pathway. It plays an important role in maintaining the homeostasis of gut microbiota [27]. PGRP-LD protects the mosquito from *Plasmodium* infection through regulating gut microbiota-mediated PM formation in *Anopheles stephensi* [30]. PGRP-LB serves as a negative regulator of immune pathways in *Aedes* and *Anopheles* mosquitoes [31, 32]. PGRP-LA also participates in antiparasitic immune defenses, but the underlining mechanism needs to be further elucidated [31].

In this study, we demonstrate that the expression of *pgrp-la* is induced in the midgut in response to a blood meal. Such induction is due to the rapid proliferation of gut microbiota post-feeding. Once gut microbiota is removed by antibiotic treatment, PGRP-LA fails to initiate the synthesis of downstream immune effectors. Knocking down of *pgrp-la* in antibiotic-treated mosquitoes has no influence on the outcome of infection with *P. berghei*. These data elucidate that the anti-*Plasmodium* effect of PGRP-LA depends on the homeostasis of gut microbiota.

## Methods

### Mosquito rearing and antibiotic treatment

The *An. stephensi* mosquito (the Hor strain) was reared in the insectary at a temperature of 28 °C, relative humidity of 80% and a 12:12 h light/dark photocycle. Adults were fed on 10% sucrose solution and mouse blood. For antibiotic treatment experiment, newly emerged adult mosquitoes were orally administrated with 10% sucrose solution containing 10 U/ml penicillin, 10 μg/ml streptomycin and 15 μg/ml gentamycin daily for 3 days [20]. Then the antibiotic-treated mosquitoes and untreated mosquitoes were collected and surface sterilized. The homogenates were plated onto LB-agar to test the efficacy of antibiotic treatment.

### *Plasmodium berghei* infection

Six to eight-week-old BALB/c mice were injected intraperitoneally (ip) with 10^6^ infected RBCs with GFP-tagged *P. berghei* (ANKA). To evaluate parasitemia, tail smears were taken and stained with Giemsa (Baso Diagnostics Inc, Zhuhai, China), the number of parasites per 3000 RBCs were counted [33]. When the parasitemia reached 4–6%, the infected mice were used to feed mosquitoes that had been starved overnight; the mosquitoes were then maintained at 21 °C. Unengorged mosquitoes were removed 24 h post-blood meal. Midguts were dissected and infection intensity (oocyst number) were determined microscopically at day 8 post-infection.

### Knockdown by RNA interference

PCR amplification from 606 to 1082 bp of *pgrp-la* (ASTE016413) was performed using T7-tagged primers. The plasmid eGFP (BD Biosciences, San Jose, USA) was used as a template for control dsRNA amplification. The double-stranded RNA (dsRNA) was synthesized using the MEGAscript RNA kit (Ambio, Invitrogen, Shanghai, China). Two to three-day-old mosquitoes were injected with 69 nl dsRNA (4 μg/μl) intra-thoracically using a nanoject II microinjector (Drummond, Philadelphia, USA). Mosquitoes treated with an equal volume of dsGFP and distilled water were used as controls. For the antibiotic treatment experiment, dsRNAs were injected three days after antibiotic treatment. The midguts of dsRNA-treated mosquitoes were dissected two days post-treatment and knock down efficacy was examined by qPCR as previously described [30]. For the *P. berghei* infection experiment, the mosquitoes were fed on an infectious blood meal four days after dsRNAs injection.

### Total RNA extraction and reverse transcription quantitative PCR (RT-qPCR)

To evaluate transcription of related genes, total RNA was extracted from whole mosquitoes or midguts using TRIzol reagent (Sigma-Aldrich, Shanghai, China) according to the manufacturerʼs protocol. The cDNA was prepared from total RNA using the 5× All-in-One MasterMix (with AccuRT Genomic DNA Removal Kit; ABM, Shanghai, China). Quantitative PCR was performed using SYBR Green qPCR Master Mix (Bimake, Shanghai, China) according to a previously described protocol [30]. Ribosomal gene *s7* was used as the internal reference gene.

### Gut microbiota measurement

For CFU measurement, midguts were dissected and collected in 0.9% NaCl 5 days after dsRNA treatment and homogenized. The homogenates were plated on LB agar plates. The CFUs were counted 2 days after incubation at 28 °C. For total gut microbiota measurement, total DNA of midguts treated with dsRNA were extracted using the Holmes & Bonner [34] method at 2 time points, 5 days post dsRNA injection and 24 h post-infectious blood meal. Bacterial density was quantified by qPCR using universal *16S* rRNA primers [35] (Additional file 1: Table S1).

### Peritrophic matrix analysis

For measurement of mRNA levels, 3 PM synthetic genes [*peritrophin1* (ASTE010406), *peritrophin14* (ASTE009456), *chitin synthase* (ASTE007145)) and 2 chitinases (*chitinaseA* (ASTE005630) and *chitinaseB* (ASTE000328)] were quantified by qPCR using specific primers (Additional file 1: Table S1) 24 h, 36 h and 45 h post-infectious blood meal. The significance was determined using the Student’s t-test. For western blot analysis, 10 midguts were dissected 24 h post-infectious blood meal. Proteins were extracted in cracking buffer (8 M urea, 2% SDS, 5% β-mercaptoethanol and 125 mM Tris-HCl). Immunoblotting was carried out using rabbit anti-per1 antibody (1:1000) and mouse anti-β-actin antibody (1:2000) (Abbkine, Beijing, China). To generate Per1 rabbit polyclonal sera, recombinant Per1 (recPer1) was amplified using specific primers (Additional file 1: Table S1) corresponding to 55–462 bp of *peritrophin1*, and expressed in pET-42a using One step cloning (C112; Vazyme Biotech, Nanjing, China). The recPer1 was purified through Ni-NTA Superflow resin by the AKTA Explorer system (GE Healthcare, Shanghai, China) and was used to generate the antibody commercially (GL Biochem, Shanghai, China). For PM structure analysis, the abdomen of *An. stephensi* was fixed at 45 h post-infectious blood meal and sectioned at 5 μm using the paraffin sectioning method and stained with Periodic Acid Schiff (PAS) (Sigma-Aldrich) as previously described [36]. The slides were examined under bright field illumination of a Nikon ECLIPSE IVi microscope connected to a Nikon Digital Sight DS-U3 digital camera.

### Statistical analysis

All statistical analyses were performed using GraphPad Prism version 6 (GraphPad Software, La Jolla, CA). Statistics of gene expression were tested for using the Student’s t-test. The Mann–Whitney test was used to determine the significance of infection rate and microbiota level of dsRNA-treated mosquitoes.

## Results

### PGRP-LA is a sensor of gut microbiota

**Fig. 1:**
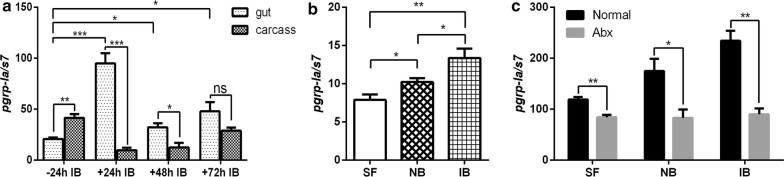
Gut microbiota is required for the expression of PGRP-LA. **a** Relative gene expression of *pgrp-la* in the midgut and carcass of mosquitoes before (− 24 h IB) and after (+ 24 h IB, + 48 h IB and + 72 h IB) feeding with infectious blood meal. **b** Relative gene expression of *pgrp-la* in midgut of sugar-fed (SF), 24 h post normal blood-fed (NB) and infectious blood-fed (IB) mosquitoes. **c** Relative gene expression of *pgrp-la* in normal and Abx midguts of sugar-fed (SF), 24 h post normal blood-fed (NB) and infectious blood-fed (IB) mosquitoes. Error bars indicate standard error of the mean (*n* = 8). Results from one of three independent experiments are shown. Significance was determined by Student’s t-test (for details, see Additional file 3: Text S1). *P < 0.05, **P < 0.01, ***P < 0.001, ns, not significant

We first analyzed spatial expression pattern of *pgrp-la* in the midgut and the remaining carcass of *An. stephensi* 24 h prior to (− 24 h) and 24, 48 and 72 h post-infectious blood meal. It was expressed abundantly in the carcass before blood-feeding. However, *pgrp-la* was upregulated significantly in the midgut within 48 h after the mosquito took an infectious blood meal (Fig. 1a). To investigate whether the upregulation of *pgrp-la* in the midgut is due to blood-feeding or the presence of parasite, we compared expression of *pgrp-la* in the midguts of mosquitoes fed on normal blood and blood containing *Plasmodium*. A blood meal, no matter whether it contained *P. berghei* or not, significantly increased the expression of *pgrp-la* comparing to sugar-fed mosquitoes (Fig. 1b). In addition, the presence of the *Plasmodium* parasite further induced the expression level of *pgrp-la* significantly than normal blood did. Because blood-feeding causes an extreme bloom of gut microbiota [9], we next hypothesized that upregulation of *pgrp-la* in response to a blood meal could be due to the proliferation of gut microbiota. Then we compared *pgrp-la* expression in the midgut of normal and antibiotic-treated (Abx) mosquitoes. As expected, once the gut microbiota was removed by antibiotic treatment, the expression of *pgrp-la* in the midgut significantly decreased, compared to control mosquitoes (Fig. 1c). Blood meal alone failed to increase the expression of *pgrp-la* (Fig. 1c). Taken together, these results indicate that the induction of *pgrp-la* expression in response to blood-feeding, is a result of the proliferation of gut microbiota in the midgut.

### PGRP-LA regulates the homeostasis of gut microbiota

**Fig. 2:**
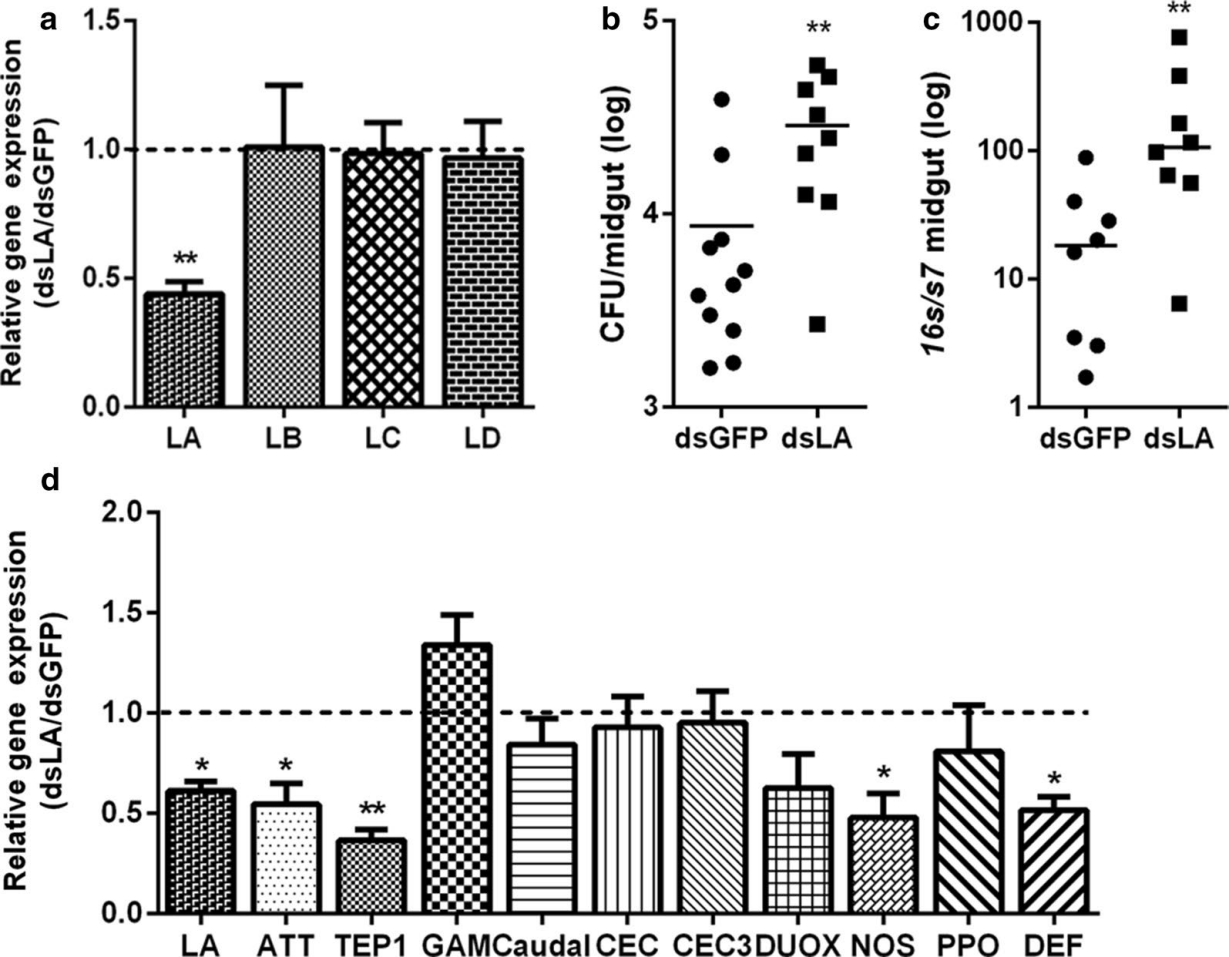
Influence of PGRP-LA on gut microbiota in *An. stephensi*. **a** PGRP-LA silencing efficiency and specificity. Relative expression level of *pgrp-la* in dsLA mosquitoes was normalized to the gene’s expression in dsGFP controls. Error bars indicate standard error of the mean (*n* = 10). Results from one of three independent experiments are shown. Significance was determined by Student’s t-test (*t*_(8)_ = 3.431, *P* = 0.0089). **b** Median culturable gut microbiota in dsRNA-treated mosquitoes. Each dot represents an individual mosquito and horizontal lines represent the medians. Significance was determined by Mann–Whitney test (*U* = 15.00, *P* = 0.0074). **c** Relative expression level of *16S* in dsRNA-treated mosquitoes. Each dot represents an individual mosquito and horizontal lines represent the medians. Significance was determined by Mann–Whitney test (*U* = 7.00, *P* = 0.0070). **d** Relative expression levels of immune-related genes in dsRNA-treated, sugar-fed mosquitoes normalized to the gene’s expression in dsGFP controls. Error bars indicate standard error of the mean (*n* = 8). Results from one of three independent experiments are shown. Significance was determined by Student’s t-test (for details, see Additional file 3: Text S1). *P < 0.05, **P < 0.01

Given that PGRP-LA senses gut microbiota, we next examined whether it could be involved in regulating the homeostasis of gut microbiota. PGRP-LA-specific double-stranded RNA (dsLA) was injected intrathoracically into *An. stephensi*. In addition to dsGFP controls, we also selected the buffer solution for dsRNA as a blank control to confirm that there was no intrinsic deficit of exogenous RNA (Additional file 2: Figure S1a). The mRNA level of *prpg-la* reduced by 58% 2 days post-dsLA treatment compared to dsGFP controls. Such treatment did not influence the expression of other long PGRPs (PGRP-LB, PGRP-LC and PGRP-LD) (Fig. 2a). Knock down of PGRP-LA resulted in an around 6-fold increase in bacterial CFU and *16S* rRNA gene copy number per midgut, respectively, compared to that in dsGFP controls (Fig. 2b, c). As PGRP-LA is the receptor of the Imd pathway in insects [31, 37, 38], we next analyzed whether the increase of gut microbiota could be due to the downregulation of immune effectors when PGRP-LA was knocked down. A total of 10 immune related genes were analyzed in mosquitoes 4 days post-dsRNA treatment. These genes include 5 antimicrobial peptides (*attacin*, *cecropin*, *cecropin 3*, *gambicin* and *defensin*), 1 negative regulator of IMD signaling pathway (*caudal*) and 4 proteins related to cellular and epithelial immune responses (*tep1*, *ppo*, *nos* and *duox*). As expected, the expressions of four genes, *attacin*, *tep1*, *nos* and *defensin* were significantly decreased in dsLA-treated mosquitoes comparing to those in dsGFP (Fig. 2d). Given the bactericidal effect of these antimicrobial peptides, our results indicate that PGRP-LA controls the abundance of gut microbiota through regulating the synthesis of downstream immune effectors.

### PGRP-LA regulates immune defense against *P. berghei*

**Fig. 3:**
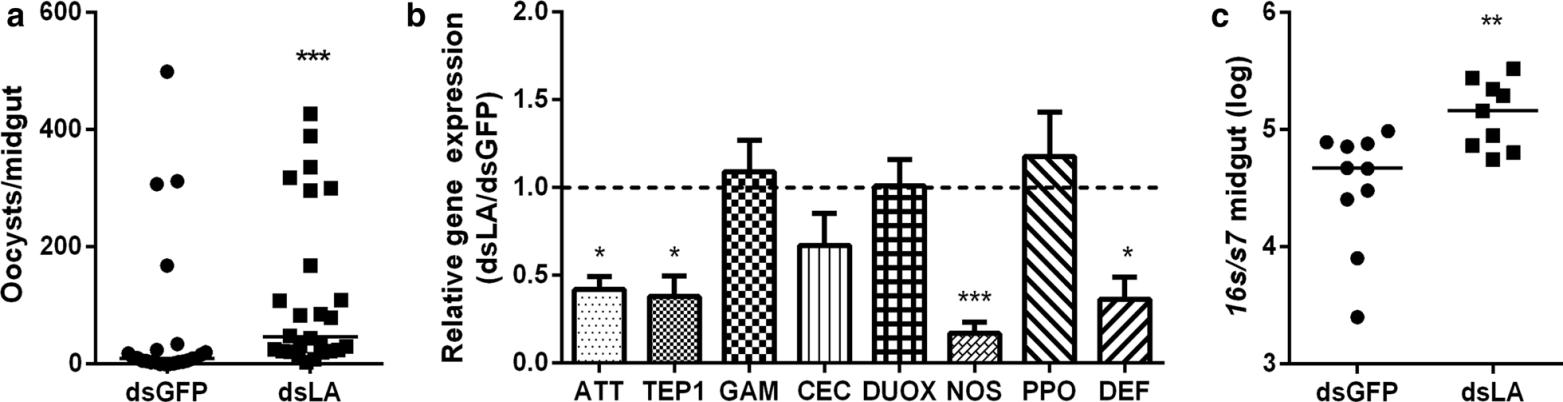
PGRP-LA is required for resistance to *P. berghei*. **a** Median oocyst number in dsRNA-treated mosquitoes. Each dot represents an individual mosquito and horizontal lines represent the medians. Results from one of three independent experiments are shown. Significance was determined by Mann–Whitney test (*U* = 132.00, *P* = 0.0003). **b** Relative expression levels of immune-related genes in dsRNA-treated mosquitoes 24 h post-infectious blood meal. Error bars indicate standard error of the mean (*n* = 8). Results from one of three independent experiments are shown. Significance was determined by Student’s t-test (for details, see Additional file 3: Text S1). *P < 0.05, **P < 0.01, ***P < 0.001. **c** Relative expression level of *16S* in dsRNA-treated mosquitoes 24 h post-infectious blood meal. Each dot represents an individual mosquito and horizontal lines represent the medians. Significance was determined by Mann–Whitney test (*U* = 12.00, *P* = 0.0057)

Given the role of PGRP-LA in regulating immune activity and its anti-parasitic function in *An. coluzzii*, we next investigated the influence of PGRP-LA on parasite defense in *An. stephensi* [31]. Knocking down of *pgrp-la* (dsLA) resulted in a significant increase in the infection rate of *P. berghei* from 9 oocysts in dsGFP to 46 oocysts in dsLA mosquitoes (Fig. 3a). Such increasing susceptibility to parasite infection was due to the downregulation of immune genes in the absence of *pgrp-la*. The four genes, *attacin*, *tep1*, *nos* and *defensin* were again downregulated in the absence of *pgrp-la* (Fig. 3b). Similarly, the downregulation of immune effectors in dsLA-treated mosquitoes facilitated the proliferation of gut microbiota as the *16S* rRNA gene was significantly higher in dsLA compared to dsGFP mosquitoes (Fig. 3c). To examine the influence of dsRNA treatment on mosquito blood uptake, we compared the weight of dsRNA-treated mosquitoes at 0 h and 24 h post-infectious blood meal, respectively. The weight of dsLA-treated mosquitoes was comparable to that of dsGFP mosquitoes at each time point (Additional file 2: Figure S1b). Taken together, our results suggest that PGRP-LA defends against *Plasmodium* infection in *An. stephensi* by positively regulating the immune signaling pathways.

### PGRP-LA is not involved in the regulation of peritrophic matrix synthesis

**Fig. 4:**
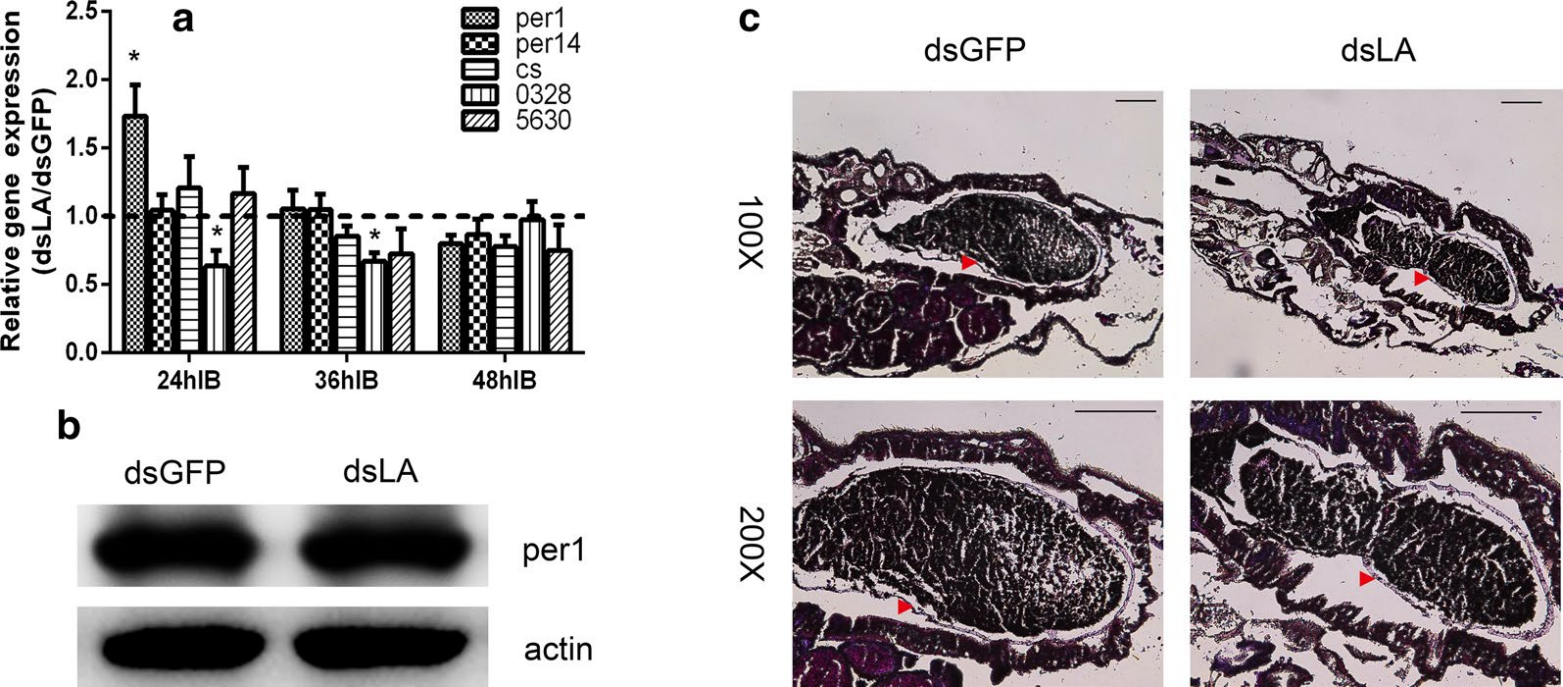
The influence of PGRP-LA on PM synthesis. **a** Relative expression levels of PM-related genes in dsRNA-treated mosquitoes 24 h, 36 h and 48 h post-infectious blood meal. Error bars indicate standard error of the mean (*n* = 8). Results from one of three independent experiments are shown. Significance was determined by Student’s t-test (for details, see Additional file 3: Text S1). *P < 0.05. **b** Western blot of Per1 in dsGFP-treated and dsLA-treated mosquitoes. Actin was used as a loading control. Results from one of three independent experiments are shown. **c** PAS staining of PM in dsGFP-treated and dsLA-treated mosquitoes at 100× and 200× magnification. Red arrowheads denote the PM. Results from one of three independent experiments are shown. *Scale-bars*: **c**, 100 μm

Gut microbiota stimulates the synthesis of peritrophic matrix (PM), thereby inhibiting *Plasmodium* infection [30, 39, 40]. As we observed an increased level of gut microbiota in dsLA mosquitoes, we next analyzed whether the increased gut microbiota in these mosquitoes could influence the integrity of PM. We first analyzed the expression level of five PM-related genes in dsRNA-treated mosquitoes 24 h, 36 h and 48 h post-infection, respectively. These genes include three PM synthesis genes (*peritrophin1*, *peritrophin14* and *chitin synthetase*) and two PM digesting chitinases [30]. Except for *peritrophin1* (*per1*) and *chitinase A* which were differentially regulated 24 h post-infection in dsLA mosquitoes, the expression of most of the genes was comparable to dsGFP mosquitoes (Fig. 4a). Then we examined the protein level of Per1 24 h post-infection by western blot and found silencing *pgrp-la* did not impact the protein level of Per1 (Fig. 4b). We further analyzed the structural integrity of PM by PAS staining. Again, no significant difference was observed between dsLA and dsGFP mosquitoes. Although knockdown of *pgrp-la* resulted in the increasing level of gut microbiota, such increase had no influence on PM structure. Neither did PGRP-LA participate in the regulation of PM synthesis directly.

### Gut microbiota is required for PGRP-LA-regulated immune defense against *Plasmodium*

**Fig. 5:**
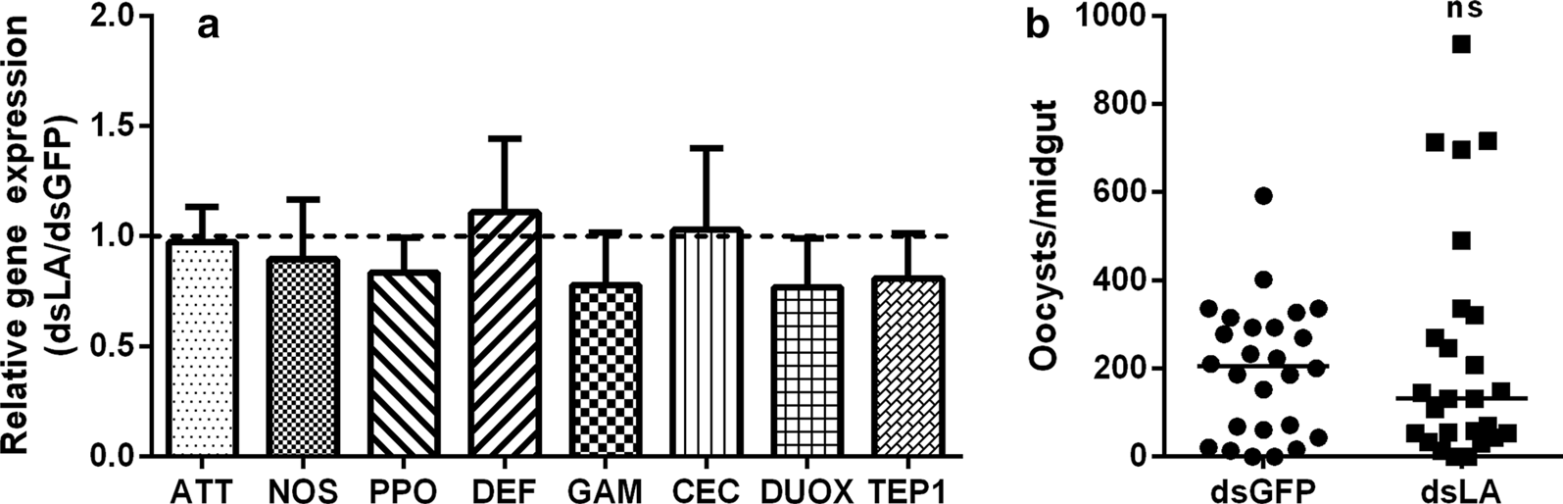
Gut microbiota is necessary for anti-*Plasmodium* of PGRP-LA. **a** Fold change of immune-related genes in antibiotic-treated mosquitoes treated with dsRNA 24 h post-infectious blood meal. Error bars indicate standard error of the mean (*n* = 8). Results from one of three independent experiments are shown. Significance was determined by Student’s t-test (for details, see Additional file 3: Text S1). **b** Median oocyst number in antibiotics-treated mosquitoes treated with dsRNA. Each dot represents an individual mosquito and horizontal lines represent the medians. Significance was determined by Mann–Whitney test (*U* = 314.50, *P* = 0.6729). *Abbreviation*: ns, not significant

As the expression level of *pgrp-la* is correlated with the abundance of gut microbiota, we next investigated whether the regulation of PGRP-LA on immune response could rely on the presence of gut microbiota. The dsRNAs were introduced into *An. stephensi* of which gut microbiota was removed by antibiotic treatment. Surprisingly, once the gut microbiota was cleared, knock down of *pgrp-la* had no impact on the expression of the immune effectors, especially *attacin*, *tep1*, *nos* and *defensin* (Fig. 5a). The infection rate in dsLA mosquitoes was comparable to that in dsGFP mosquitoes (Fig. 5b). Taken together, these results indicate that the presence of gut microbiota is required for PGPR-LA-mediated protection against infection. PGRP-LA and the regulated immune effectors in turn monitor the levels of the gut microbiota.

## Discussion

**Fig. 6:**
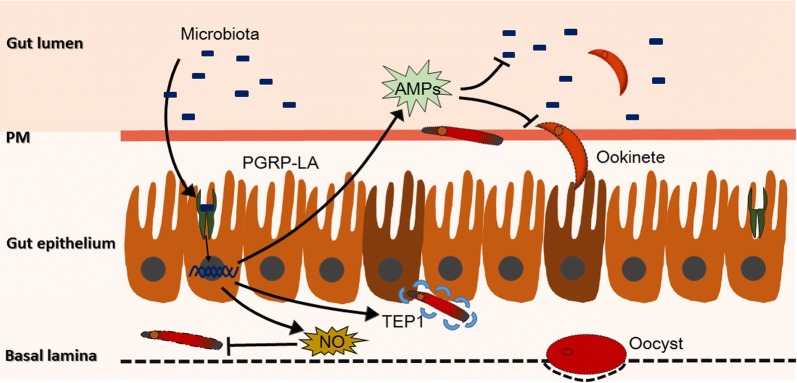
Model of influence of PGRP-LA in *An. stephensi*. PGRP-LA is enriched in midgut in response to the proliferation of gut microbiota. It protects *Anopheles* from *Plasmodium* infection through initiating the synthesis of downstream immune effectors, including AMPs, NOS and TEP1. Such regulation requires the presence of gut microbiota

The PGRP family is functionally conserved from insects to mammals, acting as pattern recognition receptors and effectors in innate immunity [29, 41, 42]. In this study, we elucidated that gut microbiota stimulates the expression of *pgrp-la* in the midgut of *An. stephensi*, which in turn regulates the microbiota growth and parasite infection through controlling immune activity. Once gut microbiota is removed, such regulation is abolished (Fig. 6).

While PGRP-LA is present in multiple insects, its function in regulating the immune response is not fully understood. In *Drosophila*, the expression of *pgrp-la* is enriched in the barrier epithelia, but low in the fat body. It is not required for the production of antimicrobial peptides by the fat body in response to a systemic infection, while it significantly stimulates antimicrobial peptide gene expression in the trachea upon oral bacterial infection [37]. In *Tribolium castaneum*, PGRP-LA functions as a pivotal sensor of the IMD pathway *via* sensing both Gram-negative and Gram-positive bacteria. It is mainly responsible for the recognition of Gram-positive bacteria, which is contrary to what has been found in *Drosophila* and *Aedes* [32, 37, 43]. Here we show that PGRP-LA is essential in regulating the immune defense against *P. berghei*. This finding is consistent with the function of PGRP-LA in *An. coluzzii* [31]. Our study further describes the mutual regulation between PGRP-LA and gut microbiota. The proliferation of gut microbiota after blood-feeding induces the expression of *pgrp-la*, while PGRP-LA regulates the homeostasis of gut microbiota. In addition, gut microbiota is necessary for PGRP-LA-regulated immune protection against *P. berghei*.

The interactions between insect immune system and gut microbiota play vital roles in determining infection outcomes. Here we show that the regulation of immune responses by PGRP-LA relies on the presence and abundance of gut microbiota. In agreement with our findings, other PGRP family members are also involved in maintaining the homeostasis of gut microbiota. For example, PGRP-SC2 contributes to the gut immune homeostasis through limiting age-related gene, *foxo*, that induces commensal dysbiosis in *Drosophila* [44]. PGRP-LC controls the Imd activity *via* cooperating with PGRP-LC-interacting inhibitor of Imd signaling (PIMS), which is a negative regulator of *Drosophila* innate immune signaling. Such interaction helps control the homeostasis of commensal bacteria [45]. PGRP-LE also promotes the growth of the symbiotic bacteria *Wolbachia*, which in turn induces the expression of PGRP-LE in the carcass of *A. aegypti* [46]. Additionally, catalytic PGRP-LB acts as a feedback inhibitor of the Imd/Rel innate immune signaling pathway by scavenging peptidoglycan released from bacteria in multiple insects [47–49].

## Conclusions

In this study, we show that gut microbiota regulates the expression of *pgrp-la*, which in turn controls the homeostasis of gut microbiota. Such finely tuned balance is key in regulating the defense against pathogen infection. Our findings will pave the way for further understanding the tripartite interactions between the mosquito, its gut microbiota and the *Plasmodium* parasite.

## Supplementary information

**Additional file 1: Table S1.** Primers used in this study.

**Additional file 2: Figure S1. a** PGRP-LA silencing efficiency in control, dsGFP and dsLA mosquitoes. **b** The weight of dsRNA-treated mosquitoes at 0 h and 24 h post-infectious blood meal.

**Additional file 3: Text S1.** Significant results from statistical analyses in this study.

## Data Availability

All data generated or analyzed during this study are included in this published article and its additional files.

## Abbreviations

MBD: mosquito-borne disease
PM: peritrophic matrix
TEP1: thioester-containing protein 1
NO: nitric oxide
AMPs: antimicrobial peptides
ROS: reactive oxygen species
PGRP: peptidoglycan recognition proteins
PRR: pattern-recognition receptor
RT-qPCR: reverse transcription quantitative polymerase chain reaction
ip: intraperitoneally
CFU: colony-forming units
PAS: periodic acid schiff
PIMS: PGRP-LC-interacting inhibitor of Imd signaling
