# Supplementary material for: Gut microbiota is essential in PGRP-LA regulated immune protection against Plasmodium berghei infection
Source: Parasit Vectors. 2020 Jan 6;13:3. doi: 10.1186/s13071-019-3876-y (PMC6945779; doi:10.1186/s13071-019-3876-y)
Supplement: Supplementary file 1 — Additional file 1: Table S1. Primers used in this study. [file 13071_2019_3876_MOESM1_ESM.docx]

**Additional file 1: Table S1.** Primers used in this study.

| Primer name  (accession #) | Primer sequences  (F, Forward; R, reverse) | Amplicon size  (bp) | Tm  (˚C) |
| --- | --- | --- | --- |
| *PGRP-LA  (ASTE016413) | F: 5' GCGGCGGTCAATCTGTTCC3' | 477 | 60 |
|  | R: 5' GACGAAGTAGATAGCGGTGGAGAAT 3' |  |  |
| ^∆^T7-PGRP-LA | F: 5' TAATACGACTCACTATAGGGCGGCGGTCAATCTGTTCC 3' | 477 | 60 |
| (ASTE016413) | R: 5' TAATACGACTCACTATAGGGACGAAGTAGA  TAGCGGTGGAGAAT 3' |  |  |
| ^∆^T7-GFP | F: 5' TAATACGACTCACTATAGGGTCAGTGGAGAGGGTGAAG 3' | 454 | 60 |
| (BD Biosciences) | R: 5' TAATACGACTCACTATAGGCTAGTTGAACGGATCCATC 3' |  |  |
| qPGRP-LA | F: 5' ACGCAGCCATCGGTGAGC 3' | 131 | 60 |
| (ASTE016413) | R: 5' GCAGACGGACAGTGTTCGGTTT 3' |  |  |
| qS7  （ASTE004816） | F: 5' TGCGGAGCGTCGTATTCTGC 3' | 79 | 60 |
|  | R: 5' ACACAGCGGTGAGCGTTCG 3' |  |  |
| qATT | F: 5' CGCCTCACCATTGTCAAGCC 3' | 125 | 58 |
| （ASTE009529） | R: 5' GTCCGTTCCGTATCCGTCCT 3' |  |  |
| qCEC  （ASTE007106） | F: 5' GCTGCTCTTTCTCGTTGCG 3' | 98 | 60 |
|  | R: 5' CGGCACCTTCCACCTTCT 3' |  |  |
| qCEC3 | F: 5' GGTAGATGGTGCGCCCCGTT 3' | 101 | 60 |
| （ASTEI01171） | R: 5 GGTAGATGGTGCGCCCCGTT 3' |  |  |
| qGAM  （ASTE002252） | F: 5' CCGCTGTTCGTCCTCGTTCA 3' | 91 | 60 |
|  | R: 5' GCACACGGACGCCAACTTCT 3' |  |  |
| qDEF  （ASTE011281） | F: 5' CCGCCTTGAACACGCTCCT 3' | 116 | 60 |
|  | R: 5' GCTGCCGACACCGAATCCA 3' |  |  |
| qTEP1  （ASTE010227） | F: 5' CCTGGGTGCGTGGGAAAC 3' | 106 | 60 |
|  | R: 5' GCCTTGCTGTCGTTCGTGAT 3' |  |  |
| qPPO  （ASTE000587） | F: 5' TTCTGCGGTTGCGGCTGG 3' | 106 | 60 |
|  | R: 5' CGGCGTCTTGCTCGTAGTCG 3' |  |  |
| qNOS  （ASTE008593） | F: 5' CAGCGAACGGACGGCAAGCA 3' | 186 | 60 |
|  | R: 5' TGACACGACCAGCGGCAGGAT 3' |  |  |
| qDUOX  （ASTE003295） | F: 5' TCGTGAGCGTCGTCAGAAGC 3' | 116 | 60 |
|  | R: 5' CCTCACCGTCCAGCGATGC 3' |  |  |
| qCAUDAL | F: 5' TGGCGGTCTCGGCGGTCAA 3' | 200 | 60 |
| （ASTE016107） | R: 5GGTGGCTGGGATGGTGGTGGT 3' |  |  |
| q16s | F: 5' AGAGTTTGATCCTGGCTCAG 3' | 250 | 60 |
|  | R: 5' CATGCTGCCTCCCGTAGGAGT 3' |  |  |
| qPer1  （ASTE010406） | F: 5' AGCCACGGCAATGCGGTTGT 3' | 140 | 60 |
|  | R: 5' TCGGACGGTTGCAGCGGTTG 3' |  |  |
| qPer14 | F: 5' TGGTGCTCGCTACGTTCGCT 3' | 172 | 60 |
| （ASTE009456） | R: 5' TGCAAACCGCCGGGACAGT 3' |  |  |
| qAechitin synthase | F: 5' CCGCCACCACCACCACCA 3' | 124 | 60 |
| （ASTE006017） | R: 5' ACGAGCCACCGAGTCAGCAG 3' |  |  |
| qChitinaseA | F: 5' CGTCTGCTGCTGACTGCTGCC 3' | 141 | 60 |
| （ASTE005630） | R: 5' TCCAACCTGCCGTTCCCACTGT 3' |  |  |
| qChitinaseB | F: 5' AACGGGCTCGGTGGTATCAT 3' | 103 | 60 |
| （ASTE000328） | R: 5' GTGCTTCCTTGGCTGCTTCA 3' |  |  |
| qPGRP-LC | F:5' TGTGCCATCGTAGCGGTCAT 3' | 98 | 60 |
| （ASTE002618） | R:5' AGCCACTCGGTTCTCGTCAC 3' |  |  |
| qPGRP-LB | F: 5' GGCGATTGGGTTGCGGATTT 3' | 116 | 60 |
| （ASTE006009） | R: 5' CGATGTCCGAGCAGGGTGTA 3' |  |  |
| qPGRP-LD | F: 5' GGCGTGTATCTGCTGCTGCT 3' | 81 | 60 |
| （ASTE010245） | R: 5' CCAGGCGGGTCGTTCCAC 3' |  |  |
| recPer1 | F: 5' GGG ATA TCG GGG ATC CGA CGA TCG TTG TCC ACC GAT 3' | 408 | 60 |
| （ASTE010406） | R: 5' GGT GGT GGT GCT CGA GTT CCT CAC ACC CAG CTA GC 3' |  |  |
|  |  |  |  |

*: Primers for gene cloning

^∆^: Primers for dsRNA synthesis

q: Primers for real time quantitative PCR

rec: Primers for protein expression
