# Supplementary figures and images for: Gut microbiota is essential in PGRP-LA regulated immune protection against Plasmodium berghei infection
Source: Parasit Vectors. 2020 Jan 6;13:3. doi: 10.1186/s13071-019-3876-y (PMC6945779; doi:10.1186/s13071-019-3876-y)

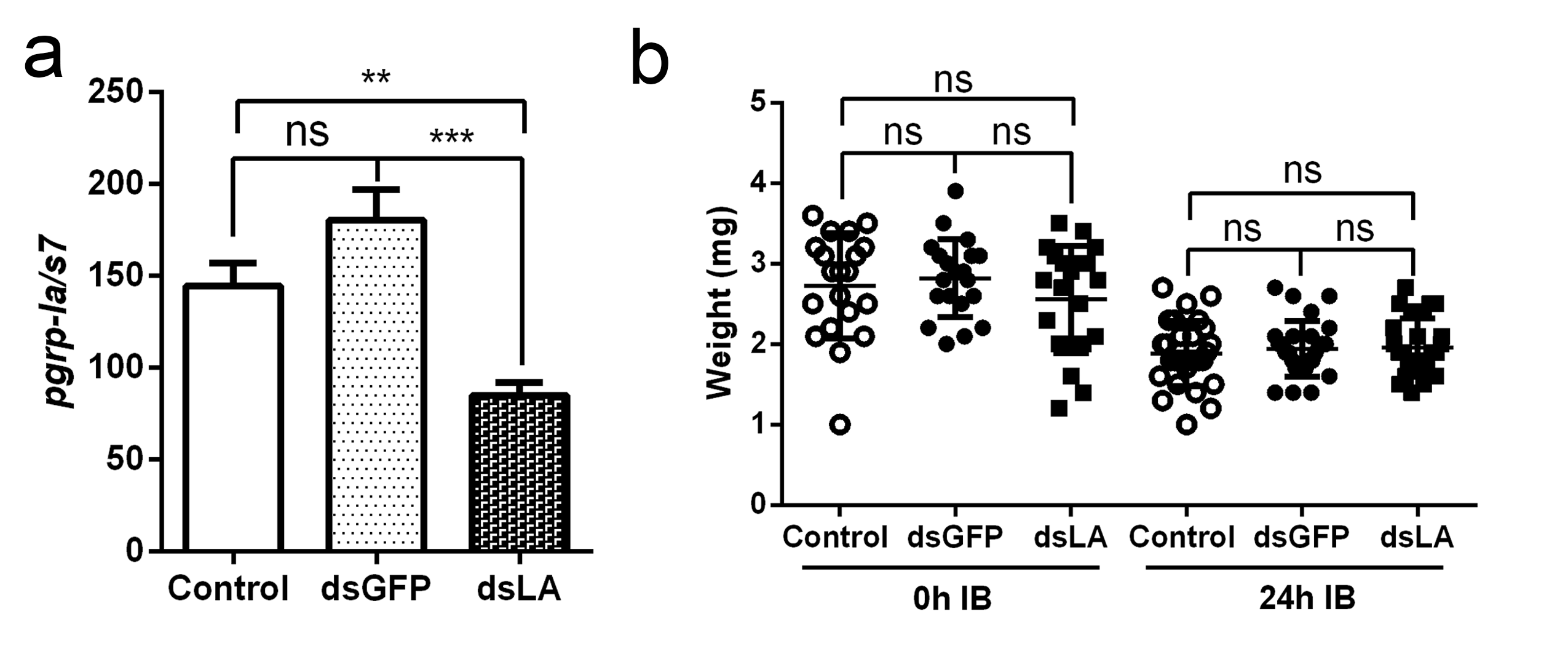

Supplement: Supplementary file 2 — Additional file 2: Figure S1. a PGRP-LA silencing efficiency in control, dsGFP and dsLA mosquitoes. b The weight of dsRNA-treated mosquitoes at 0 h and 24 h post-infectious blood meal. [file 13071_2019_3876_MOESM2_ESM.tif]
