# Supplementary material for: Gut microbiota is essential in PGRP-LA regulated immune protection against Plasmodium berghei infection
Source: Parasit Vectors. 2020 Jan 6;13:3. doi: 10.1186/s13071-019-3876-y (PMC6945779; doi:10.1186/s13071-019-3876-y)
Supplement: Supplementary file 3 — Additional file 3: Text S1. Significant results from statistical analyses in this study. [file 13071_2019_3876_MOESM3_ESM.docx]

**Additional file 3: Text S1.** Significant results from statistical analyses in this study.

Fig. 1a:

Group 1 vs Group 2: Student’s t-test: *t*_(7)_ = 4.991, *P* = 0.0016

Group 1 vs Group 3: Student’s t-test: *t*_(7)_ = 6.892, *P* = 0.0002

Group 1 vs Group 5: Student’s t-test: *t*_(7)_ = 2.587, *P* = 0.0361

Group 1 vs Group 7: Student’s t-test: *t*_(7)_ = 2.864, *P* = 0.0242

Group 3 vs Group 4: Student’s t-test: *t*_(7)_ = 7.298, *P* = 0.0002

Group 5 vs Group 6: Student’s t-test: *t*_(7)_ = 2.890, *P* = 0.0233

Group 7 vs Group 8: Student’s t-test: *t*_(7)_ = 1.897, *P* = 0.0997

Fig. 1b:

Group 1 vs Group 2: Student’s t-test: *t*_(7)_ = 2.414, *P* = 0.0465

Group 1 vs Group 3: Student’s t-test: *t*_(7)_ = 4.795, *P* = 0.0020

Group 1 vs Group 2: Student’s t-test: *t*_(7)_ = 3.164, *P* = 0.0158

Fig. 1c:

Group 1 vs Group 2: Student’s t-test: *t*_(7)_ = 4.199, *P* = 0.0040

Group 3 vs Group 4: Student’s t-test: *t*_(7)_ = 2.943, *P* = 0.0216

Group 5 vs Group 6: Student’s t-test: *t*_(7)_ = 5.019, *P* = 0.0015

Fig. 2d:

dsLA vs dsGFP

LA: Student’s t-test: *t*_(7)_ = 2.550, *P* = 0.0381

ATT: Student’s t-test: *t*_(7)_ = 3.281, *P* = 0.0135

TEP1: Student’s t-test: *t*_(7)_ = 3.878, *P* = 0.0061

GAM: Student’s t-test: *t*_(6)_ = 1.473, *P* = 0.1912

CAU: Student’s t-test: *t*_(7)_ = 0.4762, *P* = 0.6484

CEC: Student’s t-test: *t*_(7)_ = 0.06245, *P* = 0.9520

CEC3: Student’s t-test: *t*_(7)_ = 0.1169, *P* = 0.9102

DUOX: Student’s t-test: *t*_(7)_ = 0.9976, *P* = 0.3643

NOS: Student’s t-test: *t*_(7)_ = 1.930, *P* = 0.0949

PPO: Student’s t-test: *t*_(7)_ = 0.6083, *P* = 0.5622

DEF: Student’s t-test: *t*_(7)_ = 2.646, *P* = 0.0331

Fig. 3b:

dsLA vs dsGFP

ATT: Student’s t-test: *t*_(7)_ = 2.535, *P* = 0.0444

TEP1: Student’s t-test: *t*_(11)_ = 2.491, *P* = 0.0300

GAM: Student’s t-test: *t*_(7)_ = 0.9315, *P* = 0.3826

CEC: Student’s t-test: *t*_(6)_ = 0.7832, *P* = 0.4633

DUOX: Student’s t-test: *t*_(7)_ = 0.03139, *P* = 0.9758

PPO: Student’s t-test: *t*_(7)_ = 1.067, *P* = 0.3215

DEF: Student’s t-test: *t*_(11)_ = 2.361, *P* = 0.0378

Fig. 4a:

dsLA vs dsGFP

Group 1: Student’s t-test: *t*_(12)_ = 2.422, *P* = 0.0322

Group 2: Student’s t-test: *t*_(12)_ = 1.277, *P* = 0.2257

Group 3: Student’s t-test: *t*_(13)_ = 0.9849, *P* = 0.3426

Group 4: Student’s t-test: *t*_(12)_ = 2.434, *P* = 0.0315

Group 5: Student’s t-test: *t*_(14)_ = 1.105, *P* = 0.2880

Group 6: Student’s t-test: *t*_(14)_ = 0.2520, *P* = 0.8047

Group 7: Student’s t-test: *t*_(14)_ = 0.5596, *P* = 0.5846

Group 8: Student’s t-test: *t*_(14)_ = 0.7096, *P* = 0.4896

Group 9: Student’s t-test: *t*_(13)_ = 2.649, *P* = 0.0201

Group 10: Student’s t-test: *t*_(14)_ = 0.9553, *P* = 0.3556

Group 11: Student’s t-test: *t*_(12)_ = 1.761, *P* = 0.1037

Group 12: Student’s t-test: *t*_(13)_ = 0.9528, *P* = 0.3554

Group 13: Student’s t-test: *t*_(13)_ = 1.305, *P* = 0.2145

Group 14: Student’s t-test: *t*_(14)_ = 0.05781, *P* = 0.9547

Group 15: Student’s t-test: *t*_(14)_ = 0.09463, *P* = 0.9260

Fig. 5a:

dsLA vs dsGFP

ATT: Student’s t-test: *t*_(16)_ = 1.767, *P* = 0.0964

NOS: Student’s t-test: *t*_(15)_ = 0.3290, *P* = 0.7467

PPO: Student’s t-test: *t*_(16)_ = 1.008, *P* = 0.3283

DEF: Student’s t-test: *t*_(17)_ = 0.2442, *P* = 0.8100

GAM: Student’s t-test: *t*_(17)_ = 1.139, *P* = 0.2704

CEC: Student’s t-test: *t*_(17)_ = 0.6839, *P* = 0.5032

DUOX: Student’s t-test: *t*_(16)_ = 0.7631, *P* = 0.4565

TEP1: Student’s t-test: *t*_(14)_ = 0.7352, *P* = 0.4744
